# Recovery Takes Time: Loss of Signal Predicts Delayed Recovery of Vocal Cord Function After Thyroidectomy

**DOI:** 10.3390/jcm15103844

**Published:** 2026-05-16

**Authors:** Laura Guglielmetti, Sina Schmidt, Jasmin Al-Hammoud, Moritz Senne, Mirjam Busch, Joachim Wagner, Simone Harsch, Andreas Zielke, Constantin Smaxwil

**Affiliations:** 1 Departement of Surgery, Kantonsspital Winterthur, 8400 Winterthur, Switzerlandsina.schmidt@ksw.ch (S.S.); 2 Departement of Endocrine Surgery, Endocrine Center Stuttgart, Diakonie-Klinikum Stuttgart, 70176 Stuttgart, Germany; jasmin.alhammoud-hezel@diak-stuttgart.de (J.A.-H.); buschm@diak-stuttgart.de (M.B.); wagner@diak-stuttgart.de (J.W.); andreas.zielke@diak-stuttgart.de (A.Z.); 3 Departement of General, Visceral and Transplant-Surgery, University of Tübingen, 72076 Tubingen, Germany; moritz.senne@med.uni-tuebingen.de; 4 Outcomes Research Unit, Endocrine Center Stuttgart, Diakonie-Klinikum Stuttgart, 70176 Stuttgart, Germany; simone.harsch@diak-stuttgart.de

**Keywords:** thyroid surgery, vocal cord palsy, vocal cord dysfunction, time-course analysis, intraoperative neuromonitoring recurrent laryngeal nerve, thyroid cancer, thyroid nodule, multinodular goiter

## Abstract

**Background:** Post-thyroidectomy vocal cord dysfunction (PT-VCD) is an important side effect of thyroid surgery. With the introduction of intraoperative neuromonitoring (IONM), hopes have been raised that either the rate or severity of PT-VCD could be reduced. However, data to support these concepts are scarce. To better understand the relationship between IONM outcomes and the severity of PT-VCD, a detailed time-course evaluation of recovery of PT-VCD was performed in a continuous clinical quality registry from a specialized high-volume endocrine surgery center. **Methods:** Data were prospectively recorded in a single-center clinical quality assurance registry (June 2015 to May 2016) and subsequently analyzed retrospectively, with a 12-month follow-up for all cases. All patients underwent vocal cord (VC) laryngoscopy (VCL) by independent ear–nose–throat (ENT) specialists before and after surgery. Cases with newly diagnosed PT-VCD were enrolled in a detailed follow-up program (recruitment from June 2015 to May 2016) that included structured telephone interviews every 4–6 weeks to assess the exact time course of PT-VCD recovery and VC status for a period of at least 12 months. Clinical data were analyzed for variables affecting the time course of recovery by univariate analysis. **Results:** From 6/2015 to 5/2016 there were 1097 consecutive thyroid procedures. During this period, 78 cases of PT-VCD (1591 nerves at risk (NARs); 4.9%) were entered into the detailed follow-up-program. Of these, three cases of PT-VCD persisted at 12 months (PT-VCD 0.18% NAR), with six cases lost to follow-up (maximum rate of potentially persistent PT-VCD of 0.54% NAR). In total, 15% of PT-VCD cases recovered within 4 weeks; the mean recovery time was 4.4 months, and 6 months after thyroidectomy, 18% still had impaired VCL tests. Individual cases were followed >12 months showing late full recovery of PT-VCD, thereby challenging the definition of permanent VCD. Logistic regression analysis revealed non-transitory loss of signal (ntLOS) (OR for recovery within 12 weeks: 0.39; 95%CI 0.15–0.98; *p* = 0.046) and more specifically, secondary ntLOS, to be a significant independent predictor of PT-VCP recovery beyond 12 weeks (OR for recovery within 12 weeks 0.303; 95%CI 0.115–0.797; *p* = 0.016). **Conclusions:** For the first time, these data provide a detailed description of the time course of PT-VCD recovery in a large cohort, along with correlations to operative data and IONM findings. Our study indicates that recovery from PT-VCD can be prolonged, and specifically, the occurrence of ntLOS—especially secondary ntLOS—during IONM was predictive of a longer recovery trajectory. This suggests that IONM may offer an additional advantage by functioning as a prognostic tool, helping to identify patients at higher risk for extended recovery periods. Such early identification could enable a more targeted approach, potentially allowing for the earlier initiation of supportive interventions, like speech therapy, in those most likely to benefit from proactive management.

## 1. Introduction

Thyroidectomy is a commonly performed procedure for benign thyroid disorders, hyperthyroidism, and thyroid malignancies. Although generally safe, it carries a risk of postoperative complications, including hypocalcemia, cervical hematoma, and injury to the recurrent laryngeal nerve [1]. The latter may result in postoperative vocal cord dysfunction (VCD), often presenting as dysphonia and significantly impairing patients’ quality of life. VCD after thyroidectomy is therefore a feared surgical complication [2]. It is well known that its clinical course can be very different [3].

To provide transparent and comparable quality data, vocal cord tests using direct laryngoscopy and performed by an independent ear–nose–throat (ENT) specialist are essential before and after every thyroid surgery [4]. Initiation of speech therapy for post-thyroidectomy vocal cord dysfunction (PT-VCD) would benefit from a better understanding of VCD recovery. However, such data is currently not available.

In the last three decades surgical extent changed from subtotal to more radical procedures (lobectomy, total or nearly-total thyroidectomy) to prevent risky re-do operations in recurrent goiters. It became gold standard to identify and visualize the recurrent laryngeal nerve in subtile preparation and that could decrease permanent vocal cord palsies due to recurrent laryngeal nerve (RLN) injury [5].

Although intraoperative laryngeal nerve monitoring (IONM) has not been shown to reduce unilateral RLN injury, it has demonstrated benefit in preventing the dreaded complication of bilateral vocal cord palsy [6].

Numerous studies have investigated the incidence of VCD and risk factors for RLN injury following thyroid surgery, including large multi-institutional analyses and systematic reviews [7]. But, to our knowledge, there are no known clinical markers to predict the time course or risk factors indicating a late recovery of VCD. Often, there is no evident intraoperative reason for the nerve damage and in most cases the RLN keeps its macroscopic integrity [8]. Guided by this impression, surgeons tend to expect a fast recovery. Usually, speech therapy or any other special therapeutic options are postponed until protracted clinical course is observable many weeks after surgery. During this period, patients suffer from reduced voice quality and, depending on their profession, often from an inability to work. Contrary to general expectations, our follow-up data since 2015 have frequently shown a more prolonged recovery, often lasting several months.

Nowadays, more extensive preparation of the RLN and the frequent use of IONM are surgical standards and we assume that such intraoperative findings can help to understand more about the type of nerve damage and its recovery. This leads us to the current work, which aims to analyze the time course of a VCD using our follow-up data and to identify differences in IONM findings and clinicopathological markers. Predicting the expected course of a VCD recovery could help surgeons change clinical strategies to support patients with postoperative VCD and apply individualized therapeutic procedures. Therefore, we obtained a detailed time-course analysis of PT-VCD from a continuous clinical quality registry reflecting real-world experience.

## 2. Methods

### 2.1. Study Population

This study represents a retrospective evaluation of prospectively collected data from a continuous 12-month cohort of patients who underwent thyroid surgery at a specialized endocrine surgery center. The procedures were carried out by four board-certified senior surgeons. Information was obtained from the institutional registry, patient records, and the center’s standardized follow-up program. The analysis included an unselected, consecutive series of patients treated between June 2015 and May 2016. Individuals presenting with pre-existing unilateral or bilateral VCD were excluded. Data completeness was ensured for all variables recorded up to the time of hospital discharge, with no missing values identified.

### 2.2. Application of IONM and Definition of Loss of Signal (LOS) [9]

Application of IONM—the nomenclature is based on the recommendations of the International Neural Monitoring Study Group (INMSG) [10], including the recommended standards for loss of signal (LOS) evaluation/intraoperative problem-solving algorithms [11]. In primary LOS, no VC-EMG (vocal cord EMG) is obtained during surgery despite extensive efforts, including changing equipment. This means that, despite all attempts to acquire a usable EMG signal, no signal is detected. In secondary LOS, a VC-EMG is initially obtained at the vagal nerve, but the EMG signal is lost during the course of the surgery. The loss is documented as either remaining absent (non-transitory LOS: ntLOS) or recovering (transitory LOS).

### 2.3. Perioperative and Procedural Data

All patients in this cohort were treated at a high-volume referral center for thyroid and parathyroid surgery accredited by the German Association of Surgery (DGAV) [12]. All patients had pre- and postoperative vocal cord (VC) test by direct laryngoscopy (VCL) performed by an independent ENT specialist. All procedures were carried out by a board-certified endocrine senior surgeon and surgical procedures consisted of open minimal incision thyroidectomies (OMIT) via collar incisions, applying minimally invasive techniques including high-energy devices as well as standardized preparation techniques. IONM of the vagal nerve and the RLN was performed routinely in accordance with established protocols described in prior publications [13]. In case of an ntLOS, this was documented and potential reasons were recorded. Primary ntLOS was defined as no electrophysiological answer during vagal nerve stimulation at the beginning of the operation, while secondary ntLOS was defined as a LOS occurring during operation, often combined with a damaging action (e.g., traction, heat, section), providing a reason for the LOS. Structural integrity of the RLN was always checked. For special indications (e.g., re-do, malignancy, immense volume extent), continuous neuromonitoring (CIONM) was available. To avoid bilateral palsy, in cases of LOS on the first side, a change in strategy was always considered, and the resection of the second side was postponed until normal vocal cord function was proven or regained.

Postoperatively, every patient had a second VCL to document the VC function. In case of a VCD, we did not apply medical (e.g., corticosteroids) or speech therapy regularly, but patients were referred to an ENT specialist 4 weeks after surgery for further evaluation and initiation of speech therapy in case of persistence.

### 2.4. Postsurgical Follow-Up Data

All patients diagnosed with PT-VCD were enrolled in a structured and comprehensive follow-up program. This included regular, typically monthly, standardized telephone assessments conducted by the Endocrine Centers Outcomes Research Unit to evaluate patient status and the persistence of VC impairment. Follow-up continued until complete recovery was confirmed, either clinically or through laryngoscopic verification by an ENT specialist. This endpoint was intentionally selected to reflect real-world conditions, emphasizing the patient’s subjective experience and reported symptoms. In addition, all affected patients were scheduled for ENT examinations at 4 and 12 weeks after surgery.

### 2.5. Handling of Data and Statistical Analysis

All perioperative variables were collected prospectively and recorded as individual case datasets in accordance with the thyroid surgery module of the StuDoQ quality assurance registry of the German Association of Surgery (DGAV), following the acquisition of informed consent [12]. For the purpose of this analysis, all data were handled in a pseudonymized manner or evaluated in aggregated form, ensuring that no conclusions could be drawn about individual patients.

Patient characteristics were summarized using descriptive statistical methods. Continuous variables are presented either as mean values with corresponding standard deviations (SD) or as medians with interquartile ranges (IQR), depending on data distribution. Group comparisons for continuous data were performed using independent two-sample *t*-tests. Categorical variables are expressed as frequencies (%) and were analyzed using Pearson’s chi-squared test or, where appropriate, Fisher’s exact test.

To identify factors independently associated with recovery within 12 weeks after surgery, logistic regression analysis was conducted. The results of this analysis are reported as odds ratios (ORs) along with their corresponding 95% confidence intervals (CIs).

All statistical analyses were performed using SPSS software (version 25, IBM Corp., Armonk, NY, USA) and RStudio (version 3.2.1, RStudio, Inc., Boston, MA, USA). A two-sided *p*-value of less than 0.05 was considered indicative of statistical significance.

### 2.6. Ethics Approval and Consent to Participate

This investigation received approval from the Institutional Review Board of the Diakonie-Klinikum Stuttgart (IRB_002_2026) and was performed in collaboration with the Endocrine Center-certified Outcomes Research Unit. All procedures adhered to the principles outlined in the Declaration of Helsinki as well as to applicable regulatory standards. The dataset used for this analysis was derived from the StuDoQ quality assurance registry, for which prior informed consent had been secured. The present work does not constitute research involving direct participation of human subjects and does not contain any personally identifiable information. As this was a secondary evaluation conducted within the framework of quality assurance, additional informed consent was not necessary.

## 3. Results

During the 12-month period, a total of 1097 thyroid surgeries were performed. Thyroid resections comprised 538 complete resections (505 total thyroidectomies (TTX) and 33 two-staged thyroidectomies) and 559 less-than-total resections (515 hemithyroidectomies and 44 comprising central resections as well as bilateral subtotal resections such as Hartley, Dunhill, and Enderlein–Hotz), resulting in a total of *n* = 1591 nerves at risk. A total of *n* = 82 PT-VCD cases were detected, of which *n* = 4 patients were excluded due to pre-existing VCD (two tumor-related, two recurrent goiters), leaving a cohort of *n* = 78 patients for analysis.

The entire cohort including *n* = 1097 thyroid surgeries was described previously [14]. In brief, 74.5% of all thyroid patients were female, and 85.9% had benign conditions including *n* = 86 patients with Graves’ disease, all of whom underwent TTX (7.8% of the entire cohort and 15.9% of the TTX subgroup). Thyroid malignancy was found in *n* = 155 patients (14.1% of the entire cohort and 27.9% of the TTX subgroup), of whom *n* = 67 patients underwent systematic dissection of cervical lymph nodes involving Robbins regions VI and VII (6.1% of the cohort and 12.8% of the TTX subgroup). A total of *n* = 126 patients (11.5%) had undergone previous neck surgery including *n* = 54 (4.9%) operations for recurrent benign goiter.

### 3.1. Postsurgical Vocal Cord Dysfunction—Patient Characteristics

Patient characteristics of all *n* = 78 postoperative VCD patients are displayed in Table 1. The average age was 54 ± 14 years. Of these patients, *n* = 54 were female (69.2%), *n* = 34 underwent a thyroidectomy (43.6%) and *n* = 7 underwent re-do operations (9.0%). Histopathological diagnoses confirmed benign thyroid conditions in *n* = 67 (85.9%) patients and included *n* = 6 patients with Graves’ disease (7.7%). Eleven patients were confirmed to have thyroid cancer (14.1%) and a dissection of the central cervical lymph nodes had been carried out in *n* = 6 patients (7.7%). An initial presentation of these results was submitted as an abstract to the German Endocrine Surgery Congress 2022 and was accepted for oral presentation [15].

Nt-LOS of the IONM was documented in *n* = 46 cases (59%); in *n* = 10 cases a primary LOS was recorded (12.8%). Although *n* = 3 patients had a bilateral affection of VCD, none led to a tracheotomy, and two patients had a full recovery within 3 months. The third patient showed an incomplete recovery on the left side at the last VCL follow-up visit 18 months postoperatively but persistent paresis on the right side.

Complete recovery was confirmed by an ENT specialist in *n* = 50 cases (64.1%), while *n* = 14 patients (17.9%) still showed reduced VC mobility at the last ENT consultation, and *n* = 11 patients refused a follow-up visit at the ENT specialist but reported clinical recovery (14.9%). A total of *n* = 3 patients (3.8%) did not recover during the clinical follow-up.

### 3.2. Postsurgical Vocal Cord Dysfunction—Time Course of Recovery

**Figure 1:**
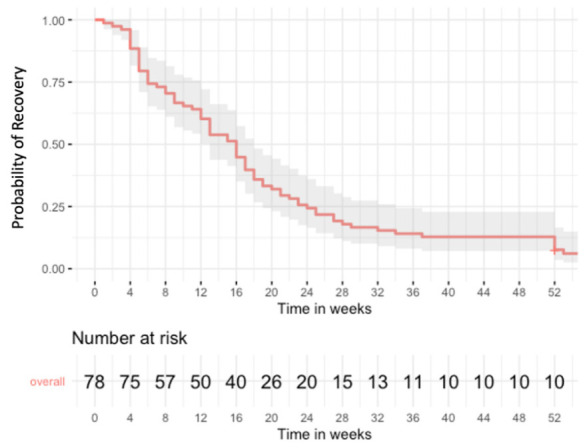
Kaplan–Meier estimates of time to recovery for all VCD patients. Shaded area = 95% confidence interval.

**Figure 2:**
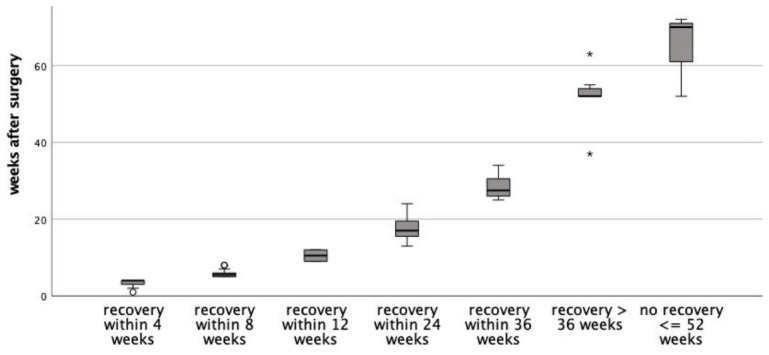
Time to recovery for all patients, stratified into 4-week intervals. The symbols ◦ and * are outliers (>1.5 IQR).

As depicted in Figure 1 and Figure 2, median recovery time was 15 weeks (IQR 6–23 weeks) for the *n* = 75 patients who recovered within the median follow-up time of 16 weeks (IQR 6–24 weeks). Median follow-up of the *n* = 3 patients without confirmed recovery within the study period was 70 weeks (IQR 61–71 weeks). The slope of the recovery curve was steepest within the 4- to 24-week post-surgery interval. After 6 months, 25.6% (*n* = 20) had not yet recovered, and *n* = 7 patients (9%) recovered later than 12 months after initial surgery. The initiation of vocal cord therapy at 4 weeks postoperatively is represented by an increasing slope after the 4-week interval.

### 3.3. Postsurgical Vocal Cord Dysfunction—Predictors for Time to Recovery

In order to further explore the possibility of clinicopathological variables to predict time to recovery, patients were stratified according to time to recovery within 12 weeks or later recovery. Table 2 shows the baseline characteristics for recovery within 12 weeks compared to later/no recovery. A total of *n* = 31 patients (39.7%) recovered within 12 weeks after surgery. Documented by VCL performed by an ENT specialist, recovery was more frequent in the group that recovered within 12 weeks (80.6% versus 53.2%; *p* = 0.017), while clinical recovery was the only documented recovery in 21.3% of the patients who recovered later than 12 weeks (3.2% versus 21.3%; *p* = 0.0042). LOS was documented in 68.1% in the later recovery group compared to 45.2% in the group of patients who recovered within 12 weeks (*p* = 0.06) and secondary signal loss was more frequent in the later recovery group (*p* = 0.02). Otherwise, the baseline characteristics were comparable between the two groups.

As depicted in Table 3, univariable logistic regression analysis revealed ntLOS and, more specifically, secondary ntLOS, as the single independent predictor of recovery later than 12 weeks (OR for recovery within 12 weeks 0.303; 95%CI 0.115–0.797; *p* = 0.016). A multivariate analysis was not feasible as only one predictor met the commonly accepted *p* < 0.2 threshold for inclusion.

## 4. Discussion

The management of PT-VCP is a critical concern in endocrine surgery, given its profound impact on patients’ quality of life. Vocal cord paralysis, especially when persistent, can lead to significant morbidity, including dysphonia, aspiration, and respiratory distress [16]. Accidental transection of the nerve is a rare occurrence. Most “injuries” to the nerve leave no visible traces and may be caused by traction or thermal affections (non-transection injury). There are few data on the recovery time of non-transection recurrent nerve injury; however, the reported duration is very heterogeneous, ranging from a few weeks to many months [17]. The variability in recovery outcomes highlights the need for a nuanced understanding of the factors influencing nerve recovery and the role of IONM [18,19]. The varying assessments of the duration of PT-VCD result in very different starting points for postoperative speech therapy in everyday clinical practice. Some clinics start immediately after surgery, while others wait a few weeks, based on the idea that many PT-VCD cases recover quite quickly [20]. Our study provides an in-depth analysis of the recovery patterns and prognostic IONM-indicators of non-transitory PT-VCD, contributing valuable insights to the existing body of literature.

One of the key findings of our study is the heterogeneity in recovery time among patients with PT-VCP. We observed that while a substantial proportion of patients (31%) regained vocal cord function within 12 weeks, a notable 9% required more than 12 months for recovery. And only a small percentage (12%) recovered within 4 weeks, with the median recovery time exceeding 4 months. This wide range of recovery times is consistent with the findings of numerous studies that emphasize the unpredictable nature of recovery from RLN injury. In a comprehensive review, Randolph et al. noted that while many patients show improvement within the first 6 months, some may experience prolonged recovery periods, with a small percentage never fully recovering [11].

The role of IONM in thyroid surgery has been a topic of considerable debate. Our study reinforces the importance of IONM, particularly in detecting LOS during surgery. We found that the occurrence of ntLOS, especially secondary ntLOS, was significantly associated with delayed recovery, underscoring the prognostic value of IONM. This finding aligns with the work of Dralle et al., who demonstrated that LOS is a strong predictor of PT-VCD [21]. Moreover, Schneider et al. reported that the use of continuous IONM to anticipate LOS during thyroid surgeries resulted in fewer cases of permanent vocal fold palsy compared with intermittent IONM [22]. And we have previously reported that IONM, when used routinely, reduces the rate of bilateral VCD in planned bilateral thyroid procedures [6]. Combining the results of our studies supports the finding that an ntLOS can predict prolonged recovery, reinforcing the recommendation to use IONM during thyroidectomy. Not only can surgery be stopped if there is LOS on the first side to avoid bilateral paresis, but LOS also provides the treatment team with information indicating that several months may be required for recovery of the first side before proceeding with surgery on the opposite side.

Interestingly, our study did not find a significant difference in recovery based on the side of RLN injury (left vs. right) or the extent of thyroidectomy (total vs. lobectomy) nor the weight of the resection specimen. This observation contrasts with some reports in the literature suggesting a higher risk of PT-VCP with right-sided injuries, possibly due to the shorter and more vulnerable course of the right RLN [23,24,25]. A study by Bergenfelz et al. suggested that unilateral paresis may be associated with older age, intrathoracic goiter or thyrotoxicosis [26], but our findings suggest that other factors, such as the extent of neural disruption as displayed by impaired neuromonitoring such as a ntLOS, may play a more significant role in determining recovery.

The initiation of VC therapy is another critical aspect of managing PT-VCP. Our study supports the early initiation of therapy, particularly in patients with documented ntLOS during surgery. Early therapeutic intervention, particularly within the first 4 to 24 weeks post-surgery, appears to be crucial for optimizing outcomes. This is consistent with findings from a study by Ruoppolo, who found that early intervention can enhance vocal outcomes, both in terms of voice recovery and quality of life [27]. The goal of treating patients with unilateral VC paralysis is to restore normal phonatory function without aspiration. Several methods have been devised to restore near-normal phonatory function, including voice therapy alone or in combination with injection medialization, laryngoplasty, or laryngeal reinnervation [28].

The clinical implications of our findings are multiple. First, the detection of ntLOS during surgery should prompt a more aggressive (vigorous) postoperative management approach, including early referral to speech therapy and close monitoring of vocal function. This proactive strategy could lead to improved patient outcomes and satisfaction, as early intervention has been shown to mitigate the long-term effects of PT-VCD. Moreover, our data support the routine use of IONM in thyroid surgery, not only as a tool to prevent bilateral PT-VCD but also as a means to provide valuable prognostic information for unilateral PT-VCD recovery. Although a few years ago IONM was not recommended as the standard of care for thyroidectomy [29], recent analyses of large registries have shown that the use of IONM is associated with a lower rate of postoperative PT-VCP [30]. One reason for this may be that the use of neuromonitoring allows for a more rapid identification of the vocal cord nerves [31]. And attempts to develop energy-based devices that also allow nerve stimulation, especially for endoscopic cervical surgery, confirm the growing understanding of the importance of nerve control [32].

Our study also highlights the need for a personalized approach to managing PT-VCP. Factors such as the presence of comorbid conditions, the extent of RLN injury, and the timing of therapeutic interventions should be considered when planning postoperative care. Since systemic conditions can significantly impact nerve healing and recovery outcomes such as diabetic neuropathy and chemotherapy-induced peripheral neuropathy, leading to impaired nerve function and regeneration [33], patients with diabetes or other systemic diseases may have a delayed or impaired healing response, necessitating a more tailored approach to their care.

It should be emphasized that although risk factors for paresis have been and continue to be investigated [34], little is known about the factors influencing the subsequent recovery process or the duration of paresis. It should also be noted that even in the absence of laryngoscopically detectable impairment of vocal cord motility, subjective voice impairment may represent a postoperative limitation for the patient. Neuromonitoring in thyroid surgery appears to improve outcomes related to baseline frequency and high-pitch voice in the early postoperative period. It could effectively reduce temporary phonation alterations after surgery [35].

In addition, the role of advanced imaging and electrophysiologic studies in assessing RLN function and predicting recovery outcomes warrants further investigation. Techniques such as transcutaneous laryngeal ultrasound for vocal cord assessment are easy to perform, non-invasive, and potentially painless alternatives for evaluating vocal cord function in selected patients [36]. And stroboscopic and intra- and extra-laryngeal electromyography have shown promise in assessing RLN integrity and guiding therapeutic decisions, including identifying the nature of postoperative recurrent laryngeal nerve dysfunction, especially in cases of normal intraoperative neuromonitoring but impaired postoperative vocal cord function [37].

Despite the strengths of our study, including a comprehensive follow-up program and the use of objective measures to assess vocal cord function, there are limitations that should be acknowledged. The retrospective design of the study and the reliance on data from a single center may limit the generalizability of our findings. And the sample size may be insufficient to draw broadly generalizable conclusions about nerve recovery.

The study also lacked comprehensive information on patient-specific factors, such as hormonal panels and body mass index, as well as granular details regarding the specific surgical procedures performed. Lastly, a broader assessment of other relevant postoperative outcomes and potential confounding side effects that could influence dysphonia and its recovery was not included, which might limit a holistic understanding of the contributing factors. Additionally, the subjective nature of patient-reported outcomes, despite being supplemented by objective assessments, introduces the potential for bias. Future research should focus on validating our findings in larger, multicenter cohorts and exploring the integration of IONM data with other clinical variables, such as patient demographics and comorbid conditions, to develop more comprehensive predictive models for PT-VCP recovery.

## 5. Conclusions

Our study contributes to the growing body of evidence supporting the importance of early detection and intervention in managing PT-VCP. The use of intraoperative neuromonitoring, particularly the detection of nt-LOS, plays a crucial role in predicting recovery outcomes and should be considered standard practice in thyroid surgery. By understanding the factors that influence PT-VCP recovery, including the role of early therapeutic interventions and the impact of systemic conditions, surgeons can better manage patient expectations and implement personalized care strategies that improve overall outcomes.

## Figures and Tables

**Table 1:** Baseline characteristics of the overall cohort.

	Overall *n* = 78
Malignant	11 (14.1)
ENT complete recovery	50 (64.1)
ENT incomplete recovery	14 (17.9)
Clinical recovery	11 (14.9)
Side of the paresis *	
- left	48 (61.5)
- right	33 (42.3)
Paresis on contralateral side	3 (3.8)
ntLOS	46 (59.0)
- primary	10 (12.8)
- secondary	36 (46.2)
Re-operation	7 (9.0)
Central lymphadenectomy	6 (7.7)
Graves’ disease	6 (7.7)
Female gender	54 (69.2)
Thyroidectomy	34 (43.6)

Abbreviations: ENT = ear–nose–throat specialist; ENT complete recovery = complete regain of the vocal cord mobility documented by an ENT; ENT incomplete recovery = markedly improved vocal cord mobility documented by a vocal cord test with a fully recovered voice, as assessed by an ENT specialist; ntLOS = non-transitory loss of signal of the intraoperative neuromonitoring. * *n* = 3 cases of bilateral paresis.

**Table 2:** Baseline characteristics for recovery within 12 weeks compared to later/no recovery.

	Recovered Within 12 Weeks *n* = 31	Delayed/No Recovery *n* = 47	*p*-Value
Malignant	3 (9.7)	8 (17)	0.511
ENT complete recovery	25 (80.6)	25 (53.2)	0.017
ENT incomplete recovery	5 (16.1)	9 (19.1)	1.00
Clinical recovery	1 (3.2)	10 (21.3)	0.042
Side of the paresis *			
- left	18 (58.1)	30 (63.8)	0.641
- right	15 (48.4)	18 (38.3)	0.483
Paresis on contralateral side	2 (6.5)	1 (2.1)	0.560
ntLOS	14 (45.2)	32 (68.1)	0.06
- primary	5 (16.1)	5 (10.6)	0.507
- secondary	9 (29.0)	27 (57.4)	0.02
Re-operation	3 (9.7)	4 (8.5)	1.00
Central lymphadenectomy	1 (3.2)	5 (10.6)	0.393
Graves’ disease	3 (9.7)	3 (6.4)	0.677
Female gender	20 (64.5)	34 (72.3)	0.617
Thyroidectomy	15 (48.4)	19 (40.4)	0.641

Abbreviations: ENT = ear–nose–throat specialist; ENT complete recovery = complete regain of the vocal cord mobility documented by an ENT; ENT incomplete recovery = markedly improved vocal cord mobility documented by a vocal cord test with a fully recovered voice, as assessed by an ENT; ntLOS = non-transitory loss of signal of the intraoperative neuromonitoring. * *n* = 3 cases of bilateral VC involvement.

**Table 3:** Univariable logistic regression of recovery within 12 weeks after thyroid surgery (*n* = 31 versus *n* = 47).

	OR	Lower CI	Upper CI	*p*-Value
Gender	0.70	0.26	1.84	0.465
Thyroidectomy vs. lobectomy	1.38	0.55	3.45	0.488
Right- vs. left-sided paresis	1.51	0.60	3.78	0.378
Graves’ disease	1.57	0.30	8.34	0.596
Thyroid cancer	0.52	0.13	2.15	0.368
Involvement of lymph nodes	0.280	0.03	2.52	0.256
Re-operation	1.15	0.24	5.54	0.860
Type of ntLOS	0.39	0.15	0.98	0.046
- primary ntLOS	1.615	0.426	6.124	0.481
- secondary ntLOS	0.303	0.115	0.797	0.016

Abbreviations: CI = confidence interval; ntLOS = non-transitory loss of signal during intraoperative neuromonitoring; primary nt-LOS = no VC-EMG obtained during surgery despite adequate stimulation efforts; secondary ntLOS = VC-EMG initially present at normal values but subsequently lost during surgery.

## Data Availability

The original contributions presented in this study are included in the article. Further inquiries can be directed to the corresponding author.
